# Dose administration time from before breakfast to before dinner affect thyroid hormone levels? 

**Published:** 2015

**Authors:** Shahram Ala, Ozra Akha, Zahra Kashi, Hossein Asgari, Adeleh Bahar, Neda Sasanpour

**Affiliations:** 1 Department of Clinical Pharmacy, Faculty of Pharmacy, Mazandaran University of Medical Sciences, Sari, Iran.; 2 Pharmaceutical Sciences Research Center, Mazandaran University of Medical Sciences, Sari, Iran.; 3 Diabetes Research Center, Mazandaran University of Medical Sciences, Sari, Iran.; 4 Student Research Committee, Mazandaran University of Medical Sciences, Sari, Iran.

**Keywords:** Levothyroxine, T4, TSH, Administration

## Abstract

**Background:**

Levothyroxine is commonly used in the treatment of patients with hypothyroidism. Levothyroxine is often administered in the morning, on an empty stomach, to increase its absorption. However, many patients have trouble for taking levothyroxine in the morning. The aim of this study was to evaluate the effect of changing administration time of levothyroxine from before breakfast to before dinner on serum levels of TSH and T4.

**Methods:**

Fifty hypothyroidism patients aged 18-75 years old were included in the study and randomly divided into two groups. Each group received two tablets per day blindly (one levothyroxine tablet and one placebo tablet) before breakfast and before dinner. After two months, the administration time for the tablets was changed for each group, and the new schedule was continued for a further two-month period. The serum TSH and T4 levels were measured before and after treatment in each group.

**Results:**

Changing the levothyroxine administration time, resulted in 1.47±0.51 µIU/mL increase in TSH level (P=0.001) and 0.35±1.05µg/dL decrease in T4 level (P=0.3).

**Conclusion:**

Changing the levothyroxine administration time from before breakfast to before dinner minimally reduced the therapeutic efficacy of levothyroxine.

Hypothyroidism is the result of inadequate production of thyroid hormone and the inadequate action of thyroid hormone in target tissues. Primary hypothyroidism is the principal cause of hypothyroidism, but other causes include central deficiency of thyrotropin-releasing hormone (TRH) or thyroid-stimulating hormone (TSH). Subclinical hypothyroidism (SCH) is present when there is a minimally elevated TSH and normal free thyroxin (FT4) level without clinical manifestation or minimal presentation (1). Hypothyroidism may be either clinical/overt, with elevation in the TSH and low levels of FT4, or subclinical, with normal levels of FT4 and elevated level of TSH. Hypothyroidism can arise as primary from the thyroid gland when there is a defect in thyroid hormone synthesis and release centrally from the hypothalamic-pituitary-thyroid axis when there is a defect in either TRH or TSH signaling to the thyroid. The condition may also be transient or permanent (1). Iodine deficiency is the most common cause of hypothyroidism worldwide. In people living in iodine-replete areas, the causes are congenital, spontaneous because of chronic autoimmune disease (primary atrophic hypothyroidism, Hashimoto’s thyroiditis) or iatrogenic due to goitrogens, drugs, or destructive treatment for hyperthyroidism (2).

Thyroid abnormalities affect considerable people of the population. However, the prevalence and pattern of thyroid disorders depend on ethnic and geographical factors most especially the iodine intake (3).

Hypothyroidism is a common endocrine disorder and is more prevalent in elderly women and in certain ethnic groups. Studies in the United States, Europe, and Japan have reported the prevalence of hypothyroidism to be between 0.6 and 12 per 1000 in women and between 1.3 and 4.0 per 1000 in men (1, 3). The National Health and Nutrition Examination Survey III (NHANES-III) data estimated the overall prevalence of hypothyroidism to be 4.6% in the American population above12 years (4). The prevalence of overt hypothyroidism was 0.3% and subclinical hypothyroidism was 4.3%. The Colorado thyroid disease prevalence survey revealed a similar prevalence of hypothyroidism of 0.4% in a self-selected group not taking thyroid hormone, but a much higher prevalence of SCH 8.5% (1, 3). In the 20-year survivor follow-up of the Wickham cohort in UK, the mean annual incidence of hypothyroidism was found to be 3.5 per 1000 in women and 0.6 per 1000 in men (5). In the large scale retreospective study in Tayside, UK between 1993-1997, the overall incidence rate of primary hypothyroidism per 1000 individuals per year was 2.97-4.98 in females and 0.88 in males. And the incidence of all causes of hypothyroidism ranged between 3.18 -3.53 per 1000 individuals per year (6). In the UK, over 23 million prescriptions for levothyroxine were written in 2010, making it the third most prescribed medication after simvastatin and aspirin (7). In a prospective study on the adult population (above 20 years) in Tehran, Iran, the incidence of overt hypothyroidism and subclinical hypothyroidism were found to be 0.28 per 1000 and 11.59 per 1000, respectively (8). 

Hypothyroidism is permanent in most patients and requires lifelong thyroid hormone replacement. Replacement with synthetic levothyroxine (LT4) is the mainstay of therapy (1, 7). Combination therapy with levothyroxine and liothyronine (triiodotyronine or T3) has been suggested as an alternative, however, the present evidence from clinical trials does not show any benefit for combination therapy compared with monotherapy with levothyroxine (9-13). Recent evidence has suggested that the dose of levothyroxine replacement is dependent on sex and body mass, but not age as it was previously thought (1, 14, 15). Many factors affect the absorption of levothyroxine; Medications such as calcium and iron compounds, aluminium hydroxide, selenium, magnesium, zinc, cholestyramine, sucralfate, raloxifene, proton pump inhibitors, and H2 blockers as well as caffeine, soybean, and fibers can impair the absorption of ingested levothyroxine (1, 7, 16, 17). Phenytoin, carbamazepine, phenobarbital, and rifampicin can increase the clearance of levothyroxine (7). Thus, it should be taken on an empty stomach without other medications, supplements, or food for 1 hour or in a similar fashion 4 hours after the last meal. A fasting regimen of administration helps to ensure that the TSH remains within a narrow target range (1, 7). The usual time schedule for taking levothyroxine tablets in patients with hypothyroidism is done every morning, before breakfast. However, some patients have difficulties in taking their medication at early morning due to gastrointestinal disturbance, forgetting the drug and pregnancy. Thus, several studies have been performed to evaluate the efficacy of the evening dose of levothyroxine (18-23). However, the literature data are inconsistent and contradictory. It has been demonstrated in the studies of Bartalena et al. (19) and Bolk et al. (20, 21) that changing the levothyroxine administration time from morning (before breakfast) to bedtime (after dinner) leads to increased absorption and increased efficacy of levothyroxine (as evident from reduced levels of TSH). On the other hand, Bach-Huynh et al. (22) reported increased serum TSH levels and reduction in serum T4 levels in response to changing the levothyroxine administration time from morning to evening. A more recent study by Rajput et al. has demonstrated equal efficacy for morning and evening doses of levothyroxine. 

The aim of this study was to investigate the effect of changing the levothyroxine administration time from before breakfast to before dinner on serum TSH land T4 levels in patients with primary hypothyroidism. This administration schedule was opted in order to evade the possibility of taking levothyroxine tablets on a full stomach as a result of short interval between dinner time and bedtime (according to the general trait in the region here, the study was conducted) and to reduce the possibility of forgetting the bedtime dose.

## Methods

**Trial design:** The present study was a prospective, randomized, double-blind, cross-over placebo controlled study. The study was approved by the Medical Research Ethics Committee of Mazandaran University of Medical Sciences and registered at Iranian Registry of Clinical Trials with registration number IRCT138903223014N2 (the full trial protocol can be accessed online at www.irct.ir ).

**Patient selection:** Patients between 18 and 75 years of both sexes, with hypothyroidism (based on a physician’s diagnosis) referring to Tuba Medical Center, Sari, Iran, were enrolled in the study. Informed written consent was obtained from all the patients. Patients with a history of gastrointestinal disorders, chronic pulmonary disorders, chronic cardiovascular disease, renal failure, diabetes, concomitant use of medications that interfere with absorption or metabolism of levothyroxine (such as cholestyramine and antibiotics and soon), and pregnant women were excluded from the study. To ensure the normal levels of TSH and T4, all patients underwent laboratory tests before the commencement of the study. In cases of TSH and T4 values above or below the normal range, the patients were subjected to levothyroxine dose adjustment and were followed-up until the normal serum levels of TSH and T4 were achieved. 

**Trial procedure:** The patients were randomly divided into two groups following a simple randomization procedure using a computer generated list of random numbers. Patients of both groups received one batch of levothyroxine and one batch of placebo and were recommended to take the tablets with a 12-hour interval (one before breakfast and one before dinner) with the predetermined dosage. The levothyroxine tablets and the placebo tablets prepared by the same manufacturer (Iran Hormone co, Tehran, Iran) were identical in shape, color and size and were packed in similar blisters. The blisters were coded with label of different colors (yellow in the case of placebo and green in the case of levothyroxine). Neither the patients nor the physicians were aware of the randomization codes until the end of the study.

The study consisted of two 60 day courses. During the first course, group A received the levothyroxine tablets in the morning, 30 minutes before breakfast, and the placebo tablet 1 hour before dinner; whereas, group B received the levothyroxine and placebo tablets in reverse order. During the second course, the levothyroxine and placebo administration times were switched within each group. The primary outcomes wherein the serum levels of T4 and TSH were measured at the end of the first and second course (on the 60^th^ and 120^th^ day of the study) using ELISA. The serum concentration values of 0.39-6.1 µIU/mL for TSH and 4.8-11.6 µg/dL for T4 were normal. 

**Statistical Analysis:** The statistical analysis of the data was carried out by SPSS Version 16. The paired- sample t-test was used to compare the data and the value of p-less than 0.05 was considered to denote a significant difference in all cases. 

## Results

**Figure 1:**
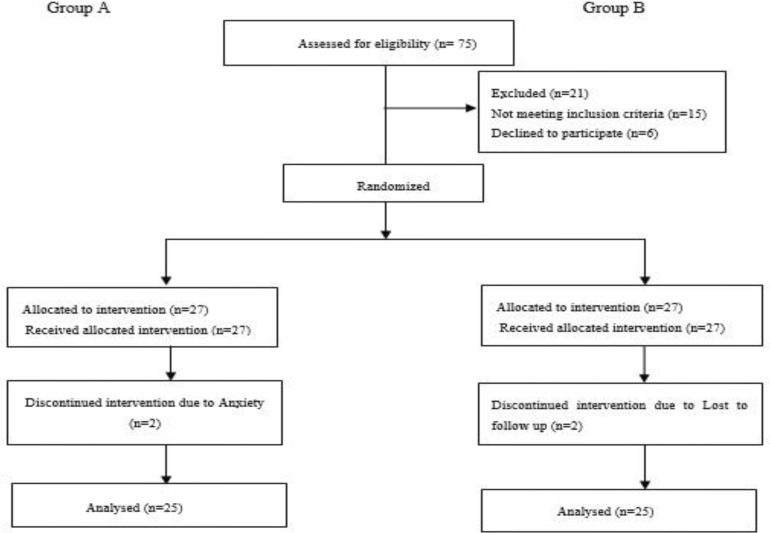
Participant flow diagram (according to guidelines of CONSORT 2010

The total number of 54 patients between 18 and 67 years were recruited in the study. Two patients (one in each arm) discontinued the study due to fear that changing the therapeutic regimen their disease might deteriorate and two patients (one in each arm) were lost to follow-up, while the 50 remaining patients were analyzed (25 patients in each group). The full participant flow diagram is depicted in figure 1.

The average administered dose of levothyroxine for the whole study population was 0.1 mg/ day. The study population was young as majority of the patients (33 or 66%) were less than 40 years (table 1).

**Table 1:** Demographic characteristics of the patients (n=50).

**Characteristic**	**Value**
Female/Male ratio	44.6
Mean age (years)	37±13.2
**Age groups** 18-30 years 30-40 years >40 years	18 (36%) 15 (30%) 17 (34%)
**Body mass index** 19-25 25-30 >30	18 (36%) 19 (38%) 14 (28%)
**Familial history of hypothyroidism** yes no	10 (20%) 40 (80%)
**Concurrent disease** None Iron deficiency anemia Hyperlipidemia Hypertension & hyperlipidemia	39 (78%) 5 (10%) 2 (4%) 4 (8%)

Fifteen (30%) patients were newly diagnosed of hypothyroidism. The most common symptoms among these patients were: dyspnea, excessive weight gain and hypersomnia. In the remaining patients, 14 (28%) patients were asymptomatic at the time of referring to a physician, 6 (12%) patients had excessive weight gain, 7 (14%) patients with dyspnea and sense of suffocation, 8 (16%) patients had weight gain, dyspnea, alopecia, hypersomnia, fatigue, restlessness and edema. 

In both groups, within group differences in the serum TSH and T4 levels as a result of changing the administration time were similar. There was significant increase in average TSH level (P_1_= 0.04, P_2_=0.035 for group A and group B, respectively), whereas, the decrease in average T4 level was insignificant (P_1_=0.7, P_2_=0.64 for group A and group B, respectively). Since the results in both crossed over groups were the same, the two groups could be regarded as one.

From the 50 patients included in the study, in 38 (76%) patients changing the levothyroxine administration time from morning to evening increased the serum levels of TSH significantly (p<0.05), and for the remaining 12 (24%) patients, the TSH levels decreased or remained constant; With regard to T4, in 33 (66%) patients, the serum levels of T4 decreased, whereas in the remaining 17 (34%) patients, the serum levels of T4 increased as a result of changing the levothyroxine administration time from morning to evening; however, the difference was not significant (p>0.05). The average values of TSH and T4 at different points during the trial are depicted in tables 2-3. 

**Table 2:** Changes to the serum levels of TSH during the study (all data are reported as Mean±SD).

**Age group (years)**	**Serum TSH (µIU/mL)**			
	**BB** 1	**BD** 2	**Difference**	**Pvalue**
≤ 40(n=33)	2.26±1.19	3.52±1.59	1.26±0.4	0.00
> 40(n=17)	1.55±1.14	2.99±1.98	1.44±0.84	0.02
Total(n=50)	2.03±1.22	3.35±1.73	1.47±0.51	0.00

1 . before breakfast,

2 . Before dinner

To investigate the effect of age on dependent variables, the data were evaluated regarding different age groups (less than 40 years and more than 40 years). There were neither any significant differences in the values of TSH or T4 between the two age groups when levothyroxine was administered before breakfast (P1=0.051 and P2=0.6, respectively) nor when levothyroxine was administered before dinner (p1=0.3 and p2= 0.1 respectively). Thus, it seems that the patients ‘age did not influence the therapeutic outcome.

**Table 3:** Changes to the serum levels of T4 during the study (all data are reported as Mean ± S).

**Age group** **Years**	**Serum T4 (µg/dL)**			
	**BB** 1	**BD** 2	**Difference**	**Pvalue**
≤ 40(n=33)	8.87±2.62	8.42±1.25	0.45±1.37	0.4
> 40(n=17)	9.20±1.62	9.05±1.25	0.15±0.37	0.7
Total(n=50)	8.98±2.32	8.63±1.27	0.35±1.05	0.3

1 . before breakfast,

2 . Before dinner

## Discussion

The current therapeutic procedure for hypothyroidism is mainly focused on hormone replacement therapy by sodium levothyroxine. The patients are usually advised to take the medication in the morning 30-60 minutes before breakfast. However, for many patients, this time schedule is not appropriate and they feel more comfortable to take the medication in the evening. In this study, the effect of changing levothyroxine administration time from morning to evening, according to serum levels of TSH and T4 was evaluated. The effects of changing the levothyroxine administration time on serum TSH and T4 levels was previously studied (19-23). But the literature data were inconsistent and contradictory.

Bartalena et al. demonstrated that greatest variation in serum concentrations of TSH are obtained when levothyroxine is administrated either early in the morning or late in the evening (19). Bolk et al. studied the effects of levothyroxine administration time (morning vs. evening) on the serum levels of TSH and T4 in 12 female patients for 4 months and found that the administration of levothyroxine in the evening results in decreased serum levels of TSH (20). However, due to the small sample size in this study, the results were not considered to be generalizable. Thus, in a more extended study by Bolk et al. later on, 105 patients were studied for a period of 6 months, with a shift in levothyroxine administration time from morning to evening and the results showed greater absorption for levothyroxine, decreased serum levels of TSH and increased levels of T4 when levothyroxine was administered at bed time (21). Bach-Huynh et al. conducted a similar study including 105 patients for 24 weeks. Their study, in contrast, demonstrated an increase in the serum TSH levels and reduction in serum T4 levels in response to changing the levothyroxine administration time from morning to evening (22). In a more recent study, Rajput et al. have studied 152 drug-naïve patients with primary hypothyroidism for the effects of morning vs. evening administration of levothyroxine on the clinical profile and quality of life. The patients were divided into two groups receiving levothyroxine either in the morning or in the evening on an empty stomach for a period of 12 weeks. The results demonstrated considerable improvement in clinical profile for majority of patients in both groups with no significance between group differences, and the evening administration of levothyroxine was reported to be as effective as the morning administration (23). 

In the present study, changing the administration time of levothyroxine before breakfast to before dinner, resulted in a considerable increase in the serum levels of TSH, in the entire study population (P= 0.001). However, the changes in serum T4 levels were insignificant and negligible (P= 0.3). This is in accordance with the results reported by Bach-Huynh et al. (22). who demonstrated a 1.25 µIU/ml increase in the average serum level of TSH as a result of changing the levothyroxine administration time form morning to evening, and Rajput et al. (23) who found equal efficacy of the two administration times, but contradict the results obtained by Bolk et al. (20, 21). This inconsistency might be in part due to the nutrition regimen in different patients and the effects of food intake on the absorption and oral bioavailability of levothyroxine. Data from screening large European population have revealed the influence of dietary iodine intake on the epidemiology of thyroid dysfunction (3).

The comparison of the TSH and T4 levels between the age groups less than 40 years and more than 40 years did not demonstrate any significant difference either in initial or the final levels of TSH and T4. In addition, there was no relationship between the BMI and the age group. Although the absorption, distribution, metabolism and excretion of levothyroxine, like any other drugs, depend on age and BMI of the patient, since the daily dose of levothyroxine was precisely determined for each patient by the endocrinologist physician on the basis of the preliminary conditions and the extent of hypothyroidism, these factors did not affect the study variables in different age groups. The study population was predominantly composed of women (88%) which reflects the fact that the prevalence of hypothyroidism is higher in women compared with men. This was also reported by Asadi et al. (8) who studied 2000 Iranian patients aged above 20 years for subclinical thyroid disorders. Their study demonstrated a higher prevalence of thyroid disorders in women compared with men (60.7% vs. 39.3% of the study population respectively), and a higher prevalence of hypothyroidism for women compared with men (11.87 per 10000 vs. 4.9 per 1000) 

Although the serum levels of T4 were not significantly changed, the changes to serum TSH levels were significant, suggesting that changing the levothyroxine administration time before breakfast to before dinner in order to enhance the patient compliance results in reduced therapeutic outcome. Nevertheless, this administration schedule may be applicable if appropriate dose adjustment is performed in order to compensate the reduced gastrointestinal absorption of levothyroxine and provide the same bioavailability of the drug.

## Notes

**Funding:** This study was funded by a grant from the Vice- Chancellery for Research of Mazandaran University of Medical Sciences and registered as Pharm.D thesis of Neda Sasanpour of the Faculty of Pharmacy.
